# Proteomic profiling of dysbiosis-challenged broilers reveals potential blood biomarkers for intestinal health

**DOI:** 10.1186/s13567-025-01570-4

**Published:** 2025-07-08

**Authors:** Svitlana Tretiak, Teresa Mendes Maia, Richard Ducatelle, Marc Cherlet, Tom Rijsselaere, Filip Van Immerseel, Francis Impens, Gunther Antonissen

**Affiliations:** 1 Livestock Gut Health Team (LiGHT) Ghent, Department of Pathobiology, Pharmacology and Zoological Medicine, Faculty of Veterinary Medicine, Ghent University, 9052 Ghent, Belgium; 2 VIB-UGent Center for Medical Biotechnology, VIB, 9052 Ghent, Belgium; 3 Department of Biomolecular Medicine, Ghent University, 9052 Ghent, Belgium; 4 VIB Proteomics Core, VIB, 9052 Ghent, Belgium; 5 Laboratory of Pharmacology and Toxicology, Department of Pathobiology, Pharmacology and Zoological Medicine, Faculty of Veterinary Medicine, Ghent University, 9052 Ghent, Belgium; 6 Impextraco NV, Wiekevorstsesteenweg 38, 2220 Heist-op-den-Berg, Belgium

**Keywords:** Blood biomarkers, dysbiosis model, gut health, broiler chicken, intestinal permeability, proteomics

## Abstract

The intestinal microbiome forms a dynamic ecosystem whose balanced composition and functioning are essential for maintaining overall gut health and well-being in living organisms. In broilers, dysbiosis disrupts the microbiota-host balance, often without obvious clinical symptoms but with intestinal inflammation, and leads to impaired animal performance. This study aimed to identify host blood-based protein biomarkers that indicate intestinal inflammation and intestinal barrier dysfunction. Using mass spectrometry-based proteomics, blood plasma samples from broilers derived from an in vivo dysbiosis model were analyzed and compared to healthy controls. Microscopic histologic changes in the gut (shortened villi, increased crypt depth) were observed in the duodenal and jejunal tissue of 25-days old challenged birds. Elevated levels of permeability markers faecal ovotransferrin and serum iohexol additionally indicated increased intestinal leakage in the challenged group. The blood plasma proteome analysis enabled quantification of 388 proteins, 25 of which were significantly different between the tested groups. The challenge was marked by activation of immune and signaling pathways, and response to bacteria, while proteins related to cellular physiology, cell–cell communication, and extracellular matrix (ECM) processes were suppressed. Protein–protein interaction analysis revealed two clusters of downregulated proteins involved in ECM organization and cell adhesion. Intestinal dysbiosis in broilers demonstrated that the host prioritizes immune defense over structural maintenance. The activation of immune processes and suppression of ECM pathways highlight potential biomarkers and therapeutic targets. Data are available via ProteomeXchange with identifier PXD056546.

**Supplementary Information:**

The online version contains supplementary material available at 10.1186/s13567-025-01570-4.

## Introduction

The functioning of the gut is largely influenced by the composition of the associated gut microbiome, as well as bidirectional interactions between the latter and the host [1]. In the physiology of the chicken, the makeup of the commensal microbiome within the gastrointestinal tract plays an essential role in modulating immunological and metabolic processes [2].

Intestinal health is vital for the welfare and performance of poultry. Enteric diseases that compromise the structural integrity of the gastrointestinal tract can result in significant economic losses, including reduced weight gain, poor feed conversion efficiency, increased mortality rates, and higher medication costs [3]. Dysbiosis refers to an alteration of compositional and metabolic activity of the gastrointestinal microbiota, i.e. an imbalance between beneficial and harmful bacteria, and is associated with performance losses but with no obvious clinical symptoms [4, 5]. Together with other stressors, dysbiosis causes disruption of the tight junctions between epithelial cells, resulting in gut leakage, villus atrophy, a decrease in nutrient absorption, and inflammation [6]. Dysbiosis became more prevalent since the complete ban on antimicrobial growth promoters (AGPs) in animal feed within the EU dated January 1, 2006 (Regulation 1831/2003/EC) [6, 7].

Although no universal microbial signature of dysbiosis exists, certain microbial patterns have been identified as indicative of dysbiosis in both laboratory animals and humans [6, 8–11]. In general, the increase in the phylum *Proteobacteria*, which includes many opportunistic pathogenic bacteria, was shown to correlate with a pro-inflammatory cytokine profile and is considered a microbial indicator of epithelial dysfunction and intestinal inflammation [9, 12].

While a direct link between intestinal inflammation, dysbiosis and loss of intercellular junction integrity in broilers has been reported by many, yet little-to-no data on the fluctuations of the blood plasma proteome under compositional changes of the intestinal microbiome in broilers is available.

Discovering new biomarkers for alterations of poultry gut health could provide new tools enabling the monitoring of gut health, as well as provide new insights into poultry physiology and pathology. Therefore, in this study, a mass spectrometry (MS)-based proteomics method was employed to investigate proteomic changes in the blood plasma of broiler chickens dysbiosis model. The aim was to identify a biomarker, or a set of biomarkers, that could serve as early indicators of dysbiosis-associated gut damage.

## Materials and methods

### In vivo dysbiosis experiment

An experimental in vivo dysbiosis challenge model was used as previously described by De Meyer et al. [5].

Briefly, a total of 96 day-old broiler chicks (Ross 308) were randomly assigned to challenge (Ctrl+) and control (Ctrl−) groups, and allocated in a pen-based housing with six birds in each pen (8 pens per group). The sample size was determined using the G*Power software (ver. 3.1.9.2, Kiel, Germany) with applied parameters of α = 0.05 and power = 95%.

Access to feed and water was provided ad libitum. All animals were fed commercial feed until day 12, after which the diet was changed to a wheat-based feed (57.5% wheat) supplemented with 5% rye. From day 12 to day 18, animals in the challenge group were administered 10 mg/kg_BW_ of florfenicol (Flordofen, Dopharma Research BV, Raamsdonkveer, The Netherlands) and 10 mg/kg_BW_ of enrofloxacin (Enroshort, Dopharma Research BV, Raamsdonkveer, The Netherlands) daily through the drinking water.

Following the course of antibiotics, during three consecutive days each bird from the challenge group received 1 mL of bacterial suspension consisting of *Escherichia coli* (G.78.71), *Enterococcus faecalis* (G.78.62), *Lactobacillus salivarius* (LMG22873), *Lactobacillus crispatus* (LMG49479), and netB- *Clostridium perfringens* (D.39.61) administered by oral gavage (Table 1). On days 19 and 20 animals were additionally challenged with a coccidial suspension containing *Eimeria acervulina* (6 × 10^4^ oocysts/mL) and *Eimeria maxima* (4.2 × 10^4^ oocysts/mL) via oral gavage. The composition of the bacterial suspension is detailed in Table 1. Strains of Eimeria were produced by Poulpharm BVBA (Izegem, Belgium).

**Table 1:** **Composition of the bacterial suspension for oral challenge**

Strain	Culturing conditions	Day 19 (CFU/mL)	Day 20 (CFU/mL)	Day 21 (CFU/mL)
*E. coli*	Luria–Bertani broth (LB, Oxoid) at 37 °C under aerobic conditions	2.9 × 10^10^	3.7 × 10^9^	3.7 × 10^9^
*L. salivarius*	Man–Rogosa–Sharpe (MRS, Oxoid) medium; microaerophilic conditions (5% O_2_)	2.39 × 10^8^	3.4 × 10^8^	4.58 × 10^10^
*L. crispatus*		2.18 × 10^8^	3.4 × 10^9^	9 × 10^8^
*C. perfringens*	Brain Heart Infusion (BHI, Sigma, Belgium) broth at 37 °C; 80% N_2_, 10% CO_2_ and 10% H_2_	2.41 × 10^9^	1 × 10^9^	2.46 × 10^9^
*E. faecalis*	Brain Heart Infusion (BHI, Sigma, Belgium) broth at 37 °C	3.9 × 10^10^	3.04 × 10^10^	2.03 × 10^10^

Broilers in the challenge group were orally inoculated with 1 mL of a bacterial cocktail consisting of *Escherichia coli*, *Enterococcus faecalis*, *Lactobacillus salivarius*, *Lactobacillus crispatus*, and *Clostridium perfringens* (netB−) on day 19, 20 and 21, with number of colony-forming units (CFU) per strain as indicated in the table.

To assess the level of intestinal permeability, a non-invasive, nontoxic marker iohexol was used as reported in other studies [13, 14]. On day 25, which is 1 day prior to euthanasia, seven animals per treatment group selected randomly were orally administered a solution containing iohexol (Omnipaque 350, GE Healthcare AS, Oslo, Norway) at a concentration of 64.7 mg/kg_BW_. An hour after administration, blood was collected from the wing vein, allowed to clot at room temperature, and, finally, was processed by centrifugation (1500 rcf, 15 min). Serum samples were collected, aliquoted and stored at −20 °C until further analysis.

**Figure 1:**
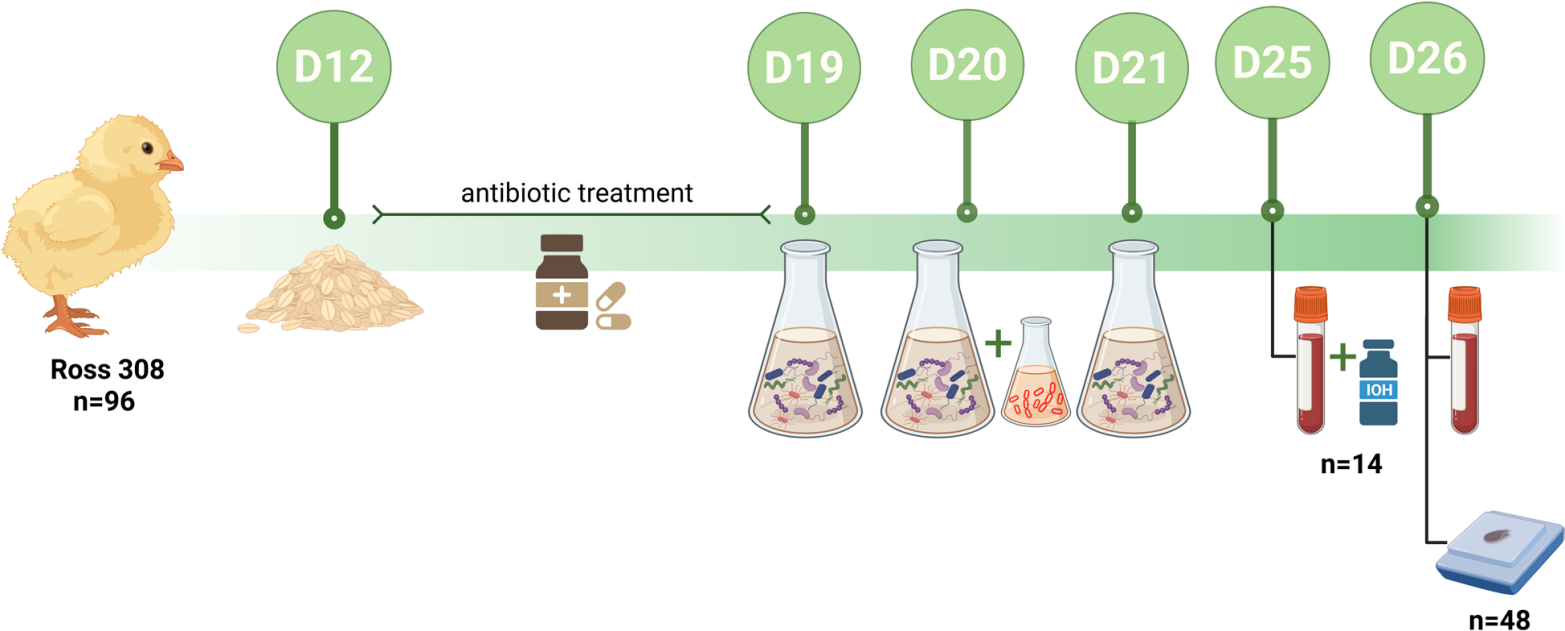
**Graphical abstract of the in vivo dysbiosis study.** A total of 96 day-old broiler chicks (Ross 308) were randomly assigned to two groups, a control and a challenge group (8 pens per treatment and 6 broilers per pen). All animals initially received commercial feed prior to transitioning to a wheat-based diet (57.5%) supplemented with 5% rye on day 12. During days 12–18, challenge animals were administered daily antibiotics (10 mg/kg_BW_ florfenicol and 10 mg/kg_BW_ enrofloxacin) via drinking water. Following antibiotic treatment (days 19–21), animals received daily oral gavage with a defined bacterial cocktail consisting of *E. coli*, *E. faecalis*, *L. salivarius* and *L. crispatus*, *C. perfringens* (netB-). On day 20, challenged animals were administered a coccidial suspension [*E. acervulina* (6 × 10^4^ oocysts/mL) and *E. maxima* (4.2 × 10^4^ oocysts/mL)]. On day 25, intestinal permeability test using permeability marker iohexol was performed. All animals were euthanized on day 26; blood was collected for protein extraction, the mid-segments of duodenum, jejunum and ileum were sampled for histological examination, content from ileum and colon was collected for the immunoassay. This figure was created with Biorender.com and exported under a paid subscription.

On day 26, prior to euthanasia, a 3 mL blood sample was drawn from each chicken (*n* = 96) via venipuncture of the jugular vein and collected into an EDTA-treated vacutainer. Plasma was then separated by centrifuging at 1900 rcf for 10 min at room temperature. The plasma samples were immediately frozen in liquid nitrogen and stored at −70 °C. Animals were euthanized with an overdose of barbiturate (sodium pentobarbital 20%, KELA Laboratoria NV, Hoogstraten, Belgium). Segments of mid-duodenum, mid-jejunum, and mid-ileum were sampled and fixed in 4% formaldehyde for histological examination. Ileal content was collected and stored at −20 °C until used for protein extraction for ovotransferrin (OVT) detection via immunoassay. A schematic overview of the in vivo experiment is presented in Figure 1.

All the experiments and manipulations involving animals were approved by the ethical committee of the Faculties of Veterinary Medicine and Bioscience Engineering of Ghent University (EC2020-045).

### Morphometrical evaluation

The segments of small intestine (*n* = 24 per group) were fixed in 4% formaldehyde for 24 h, dehydrated in xylene, and embedded in paraffin. Sections of 4 µm thickness were prepared using a microtome (HM360, Thermo Scientific, Waltham, MA, USA) and processed following the protocol outlined by De Maesschalck et al. [8]. After staining with haematoxylin and eosin, morphological parameters were evaluated using standard light microscopy. Villus length, from the crypt-villus junction to the villus tip, and crypt depth, from the junction to the base, in the mid-duodenum, mid-jejunum, and mid-ileum were determined by randomly measuring 10 villi and 10 crypts per section at 50× magnification. These measurements were done using a Leica DM LB2 microscope equipped with a camera and the LAS V4.1 computer-based image analysis software (Leica Application Suite V4, Wetzlar, Germany).

### Detection of ovotransferrin by enzyme-linked immunosorbent assay (ELISA)

The concentration of ovotransferrin was measured in the samples of ileum content as described by Goossens et al. [3]. Frozen samples (*n* = 24 per group) were thawed at room temperature. Of each sample, 150 mg was diluted in 1500 µL TBS (50 mM Tris, 150 mM NaCl, pH = 7.2) containing a protease inhibitor cocktail (P2714, Sigma-Aldrich, Merck, Darmstadt, Germany). The samples were mixed by vortexing (2 × 1 min). After centrifugation (13 000 rcf, 10 min, 4 °C), the supernatants (proteins) were collected and used in duplicate (1/50 dilution) in the ELISA (Chicken Ovotransferrin ELISA, KT-530, Kamiya Biomedical Company, Tukwila, WA, USA). The ELISA was performed according to the manufacturer’s instructions.

### Iohexol quantification in serum

Serum concentrations of iohexol were measured using ultra-high performance liquid chromatography–tandem mass spectrometry (UHPLC-MS/MS) as validated by Stroobant et al. [15].

For the analysis, serum samples (100 μL) were diluted with 100 μL of Milli-Q water and spiked with 25 μL of the internal standard iohexol-d5 (100 µg/mL). Subsequently, 15 μL of 100% trifluoroacetic acid was added. The samples were vortexed for 10 s and then centrifuged at 13 000 rcf for 15 min. The supernatant was transferred to an autosampler vial, and 5 μL was injected into the UHPLC-MS/MS instrument (Quattro Premier XE, Waters, Milford, MA, USA). A matrix-matched calibration curve and quality control samples were prepared by spiking blank serum with a known concentration of iohexol. The lowest limit of quantification for iohexol was established at 0.25 µg/mL.

### Plasma preparation for proteomics analysis

One microliter (1 µL) of each blood plasma sample, from 3 birds per pen, was added to 50 µL S-trap buffer containing 5% sodium dodecyl sulphate (SDS) and 50 mM triethylammonium bicarbonate (TEAB), pH 7.55. After protein reduction with 15 mM dithiothreitol for 30 min at 55 °C and alkylation with 30 mM iodoacetamide for 15 min at RT in the dark, phosphoric acid was added to reach a final concentration of 1.2%. The samples were diluted sevenfold with binding buffer (90% methanol in 100 mM TEAB, pH 7.1) and loaded onto a 96-well S-Trap™ plate (ProtiFi) by centrifugation at 1500 rcf for 2 min at RT. Bound proteins were washed three times with 200 µL of binding buffer, followed by centrifugation at 1500 rcf for 2 min at RT. Proteins were digested overnight at 37 °C using trypsin (1/100, w/w) in 50 mM TEAB, pH 7.55. After digestion, peptides were eluted in three steps: first with 80 µL of 50 mM TEAB, then with 80 µL of 0.2% formic acid (FA) in water, and finally with 80 µL of 0.2% FA in water/acetonitrile (50/50, v/v). The peptides were dried by vacuum centrifugation and stored at −20 °C for further analysis.

### LC–MS/MS and data analysis

Dried peptides of all samples were re-dissolved in 20 µL loading solvent A, and peptide concentration was determined on a Lunatic spectrophotometer (Unchained Labs, Pleasanton, CA, USA) as described before [16]. The concentration was adjusted to 0.015 µg/µL with 0.1% FA. Indexed retention time (iRT) peptides (P/N Ki-3002-1, Biognosys, Schlieren, Switzerland) were added to each sample according to the instructions of the manufacturer. Then, 300 ng of each sample was loaded onto Evotips (Evosep, P/N EV2003, Odense, Denmark), following the instructions of the manufacturer, except for substituting the wash step after sample loading with two washes using 80 µL 0.1% FA. All loaded Evotips were stored in 0.1% FA at 4 °C until LC–MS/MS analysis was started. Samples were analysed using an Evosep One LC-system connected in-line to a Q Exactive HF mass spectrometer (Thermo Fisher Scientific). Peptides were separated using the 15 SPD method (88 min gradient) on an endurance Evosep column (15 cm × 150 µm I.D., 1.9 µm beads, Evosep). For peptide elution, mobile phases consisted of 0.1% FA in LC–MS-grade water and 0.1% FA in ACN. The mass spectrometer operated in data-independent acquisition (DIA) mode, systematically isolating and fragmenting peptides in overlapping 10 m/z isolation windows. Full-scan MS spectra ranged from 375–1500 m/z, with an AGC target of 5 × 10^6^, maximum fill time of 50 ms, and a resolution of 60 000 at 200 m/z. This was followed by 30 quadrupole isolations with a precursor width of 10 m/z for HCD fragmentation at 30% NCE, with a target value of 3 × 10^6^ and a maximum injection time of 45 ms. MS2 spectra were acquired at a resolution of 15 000 at 200 m/z in the Orbitrap analyser without multiplexing. The Skyline software was used to create isolation intervals from 400–900 m/z, with a 5 m/z overlap. Instrument performance was monitored throughout the project using QCloud [17]. The LC–MS/MS method was described in detail earlier [18–20].

Raw files corresponding to the 48 DIA runs were searched together using the DIA-NN software (version 1.8.1) with mainly default settings [21]. Spectra were searched in library-free mode against the *Gallus gallus* canonical reference proteome sequences present in the UniProt database (database release version from June 2022), containing 18 112 sequences, with default settings. A precursor mass range filter of 400–900 m/z was applied, match between runs (MBR) option was enabled and “mass accuracy” (MS2 mass accuracy) and “MS1 accuracy” (MS1 mass accuracy) parameters were set at 20 ppm and 10 ppm, respectively. A maximum of two trypsin missed cleavages and of five variable modifications (acetylation on protein N-termini or oxidation of methionine residues) were allowed.

Further statistical analysis was conducted using an in-house script in the R programming language (version 4.1.1) [22]. Protein expression matrices were prepared by filtering the DIA-NN report table with a precursor and protein library q-value cut-off of 1%. Only proteins identified by at least one proteotypic peptide were retained MaxLFQ-normalized protein intensities were log_2_-transformed, and proteins with valid values in at least 50% of samples in one experimental condition (more than four values) were retained, resulting in 420 identified proteins. Imputation by random sampling from a normal distribution centered around the noise level (using the DEP package) was applied for missing values [23]. Pairwise comparisons between experimental groups were conducted using the limma package [24], followed by Benjamini–Hochberg method for multiple hypothesis testing. Statistical significance was set at an FDR cut-off of 0.05 and |log_2_FC|= 1, which allowed the quantification of 388 proteins.

### Functional enrichment analysis

Functional analysis aiming to identify gene ontology (GO) terms associated with dysbiosis-induced proteomic changes was performed using Gene Set Enrichment Analysis (GSEA). Using the GSEA software (version 4.2.3) [25], gene names and associated F-statistic values were given as input for a GSEA pre-ranked analysis against all Gene Ontology (GO) annotation categories: biological process (BP), cellular component (CC), and molecular function (MF). An FDR q-value cut-off value of ≤ 0. 05 for enrichment significance was applied. A total of 329 out of the 412 quantified proteins were annotated and included in the analysis. The GO gene sets were obtained from the GSEA database [25].

To gain a deeper understanding of the interactions and regulatory mechanisms of differentially abundant proteins in dysbiosis, the Search Tool for Retrieval of Interacting Genes database (STRING, version 12.0) [26] was consulted. MCL clustering algorithm in “evidence” mode with medium confidence, and the inflation parameter of 1.5 was applied.

### Statistical analysis

Statistical analysis of intestinal morphology was conducted using GraphPad Prism software (version 8.4.3, San Diego, CA, USA). The performance parameter, i.e. body weight (BW) results, were analyzed using linear model fitting, for which the association between the type of treatment (challenged or not) and body weight of individual birds was measured. The values of BW were set as fixed effect, and the data allocation of animal in the experimental facility (pen) was added as random effects. The modelling was performed within the R Studio software (version 4.1.1) using lme4 package.

Further statistical analysis for morphometric parameters, intestinal integrity, and results of the immunoassay was done using the GraphPad Prism software (version 8.4.3). First, normality of the data distribution was evaluated with a Kolmogorov–Smirnov test; an independent samples t-test or non-parametric Kolmogorov–Smirnov test was applied depending on whether the data were normally distributed or not. *P*-values for villus-to-crypt ratio comparisons in duodenum, jejunum, and ileum were adjusted using the Bonferroni’s method to correct for multiple testing.

## Results

### The challenge negatively affects intestinal integrity

**Figure 2:**
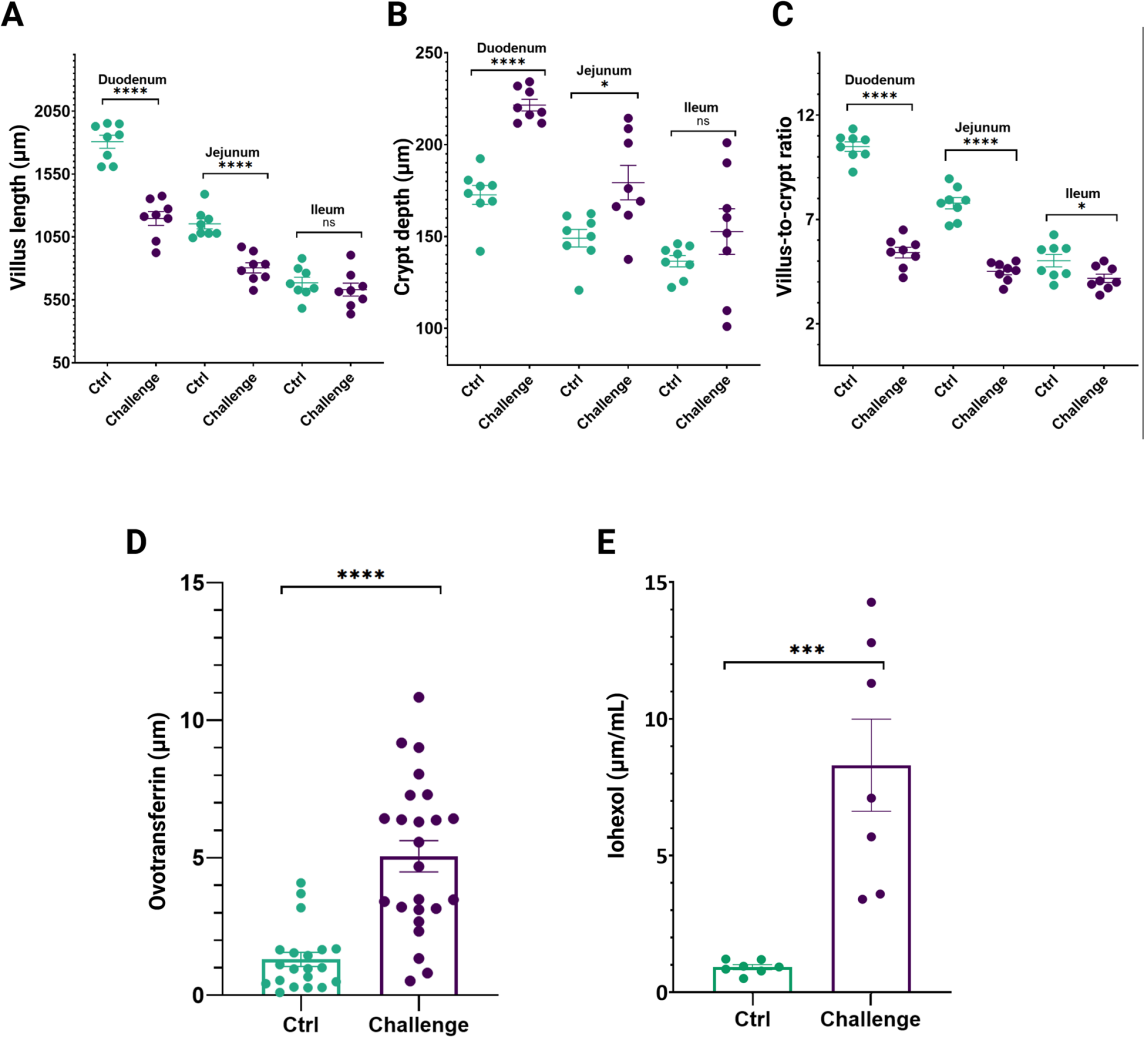
**Histological parameters in small intestinal segments and permeability markers from birds derived from a dysbiosis in vivo trial. A** Villus length evaluation measured from the crypt–villus junction to villus tip.** B** Crypt depth evaluation measured from the junction to the base. **C** Villus-to-crypt ratio. Each dot represents the mean of 3 birds per pen with a total of 8 pens for control and challenged birds. **D** Ovotransferrin concentrations in the ileum content measured by ELISA (mean ± standard error of the means) in birds from challenged and Ctrl groups (*n* = 24 animals per group).** E** Barplot representing the iohexol serum concentrations (*n* = 7 animals per group) on day 25 of the in vivo trial. Asterisks *, *** and **** correspond to significance *p*-values of *p* < 0.05, *p* < 0.01, and *p* < 0.0001, respectively; mean value ± standard error of the mean (SEM) are mentioned.

The body weights measured upon euthanasia were found significantly lower in the challenged group compared to the healthy animals (challenged (mean BW ± SEM): 1030 g ± 23.2 g, Ctrl− (mean BW ± SEM): 1134 g ± 17.2 g; *p* = 0.0008). Evaluated morphometric parameters identified significantly shorter villi (duodenum: 1197 ± 55.02 µm vs 1807 ± 51.95 µm; jejunum: 804.6 ± 40.5 µm vs 1154 ± 40.25 µm), deeper crypts (duodenum: 221.5 ± 3.16 µm vs 172.6 ± 5.14 µm; jejunum: 179.3 ± 9.35 µm vs 149 ± 4.76 µm), and a lower villus-to-crypt ratio (duodenum: 5.4 ± 0.25 µm vs 10.49 ± 0.22 µm; jejunum: 4.51 ± 0.16 µm vs 7.77 ± 0.27 µm) in the segments of duodenum and jejunum in animals from the challenge group in comparison to the control group (Figure 2A–C, respectively). In the ileum, no differences in villus length and crypt depth were recorded.

Additionally, increased intestinal permeability was confirmed by increased concentrations of intestinal permeability markers in challenged animals. Significantly higher levels of ovotransferrin (5.05 ± 0.5 µg/g vs 1.3 ± 0.25 µg/g) were detected in the intestinal content of broilers subjected to the challenge (Figure 2D); similarly, the serum concentration of the permeability marker iohexol (8.3 ± 1.68 µg/mL vs 0.9 ± 0.09 µg/mL) was higher in the challenged group (Figure 2E).

### Intestinal dysbiosis modifies blood plasma proteome

**Figure 3:**
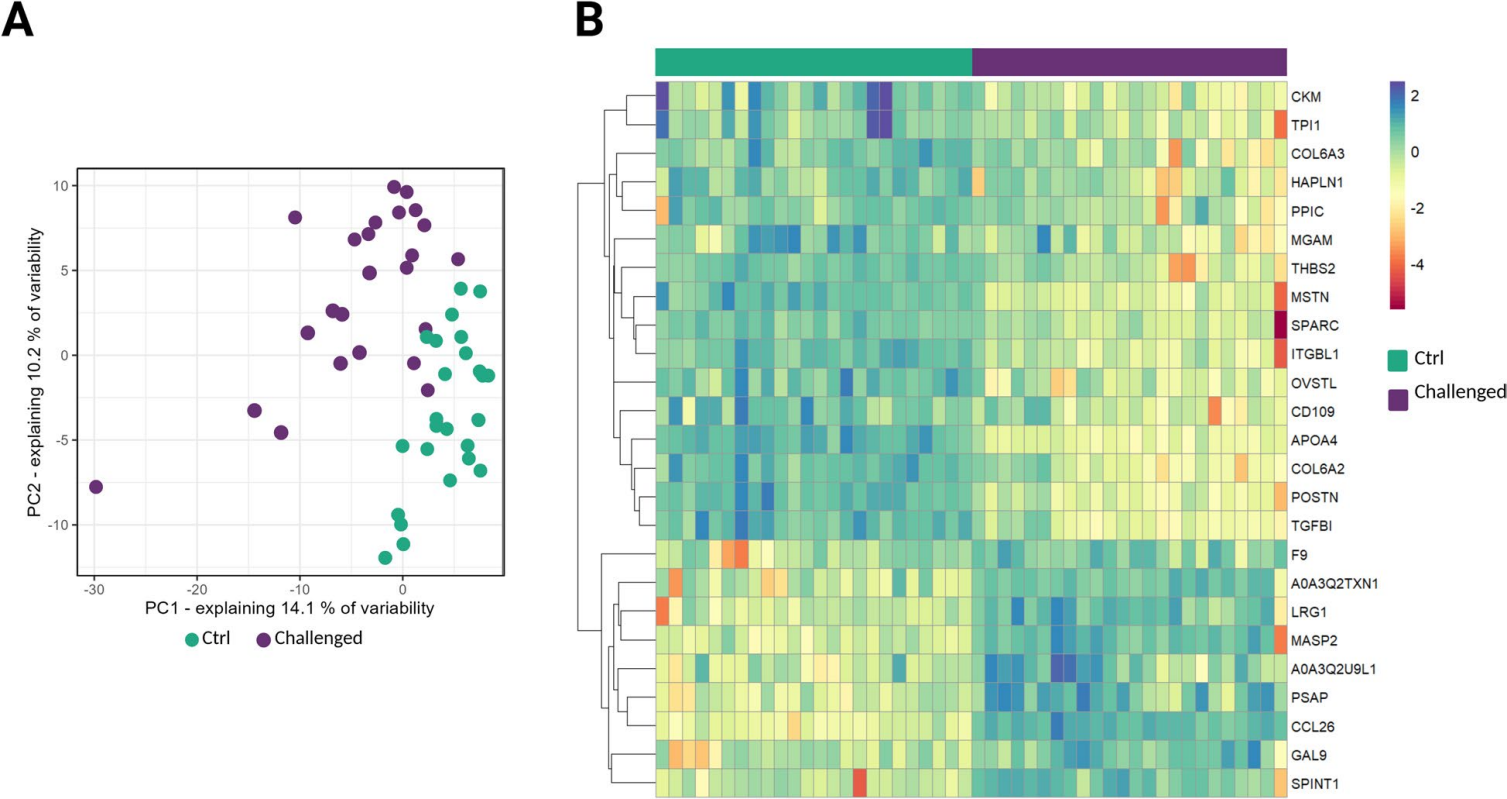
**Effects of dysbiosis challenge on plasma proteome. A** Principal component analysis (PCA) of plasma samples based on proteomic data. Plasma samples from each group were plotted along the two principal components (PCs) and grouped accordingly. All quantified proteins identified per replicate were used as variables for the PCA. The percentages of explained variance for each PC are indicated on the x and y axes. Each colored circle represents a sample (n = 48). **B** Heatmap of z-scored expression values of differentially regulated proteins (|log_2_FC|= 1; adj.p-value < 0.05) in each analyzed sample, after hierarchical clustering. The color key changes from red to dark blue to indicate the lowest to the highest protein expression.

The plasma proteome samples from Ctrl− (*n* = 24) and challenged groups (*n* = 24) were analyzed using comprehensive MS-based shotgun proteomics analysis**.** A total of 5309 peptides were detected by the MS/MS analysis and were subsequently assigned to 388 proteins. Principal component analysis (PCA) of the replicate samples, using all quantified proteins as variables, grouped samples from the same test group together, highlighting similarity in protein expression profiles (Figure 3A).

### Dysbiosis results in differentially regulated plasma proteins

Significant changes in protein levels between the control and challenged animals were explored by conducting statistical testing using limma [24]. Changes in the protein levels between challenge and control groups were assessed, including all reliably quantified proteins (*n* = 388) in the analysis (Additional file 1). This enabled the discovery of 25 differentially abundant proteins, among which 9 proteins were discovered as consistently significantly upregulated, while the other 16 proteins were significantly downregulated in the challenged animals (Figure 3B).

**Figure 4:**
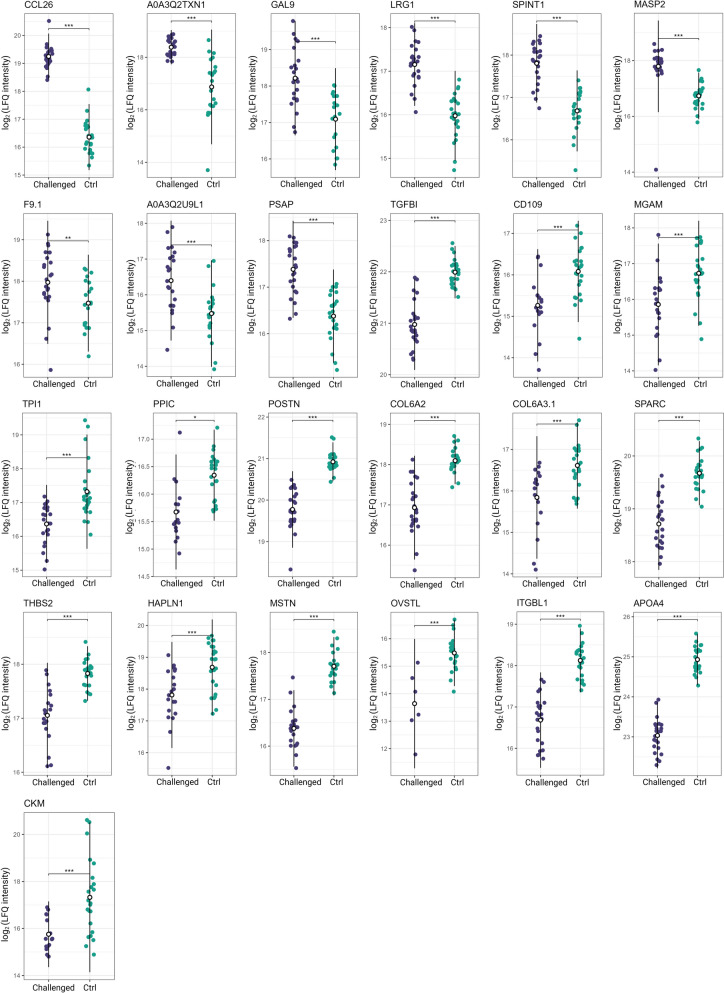
**Abundances of differentially regulated proteins detected with MS-proteomics in blood plasma of non-challenged and challenged chickens.** Each plot represents one of analyzed proteins. Each dot corresponds to one animal per experimental condition. Asterisks *, ** and *** represent statistical significance of p < 0.05, p < 0.01, and p < 0.001 between control and challenge group, respectively.

In challenged birds, levels of chemokine 26 (CCL26), complement receptor type 2-like (CR2), and gallinacin 9 (GAL9) (log_2_FC > 1.5, see Additional file 1) were affected the most. On the contrary, abundance of plasma creatine kinase M-type (CKM), apolipoprotein 4 (APOA4) and integrin subunit β-1 (ITGBL1) were reduced in the challenged birds (log_2_FC < 1.5, see Additional file 1). Abundances of differentially regulated proteins in the samples from challenged and control groups are indicated in Figure 4.

### Functional analysis of differentially abundant plasma proteins

**Figure 5:**
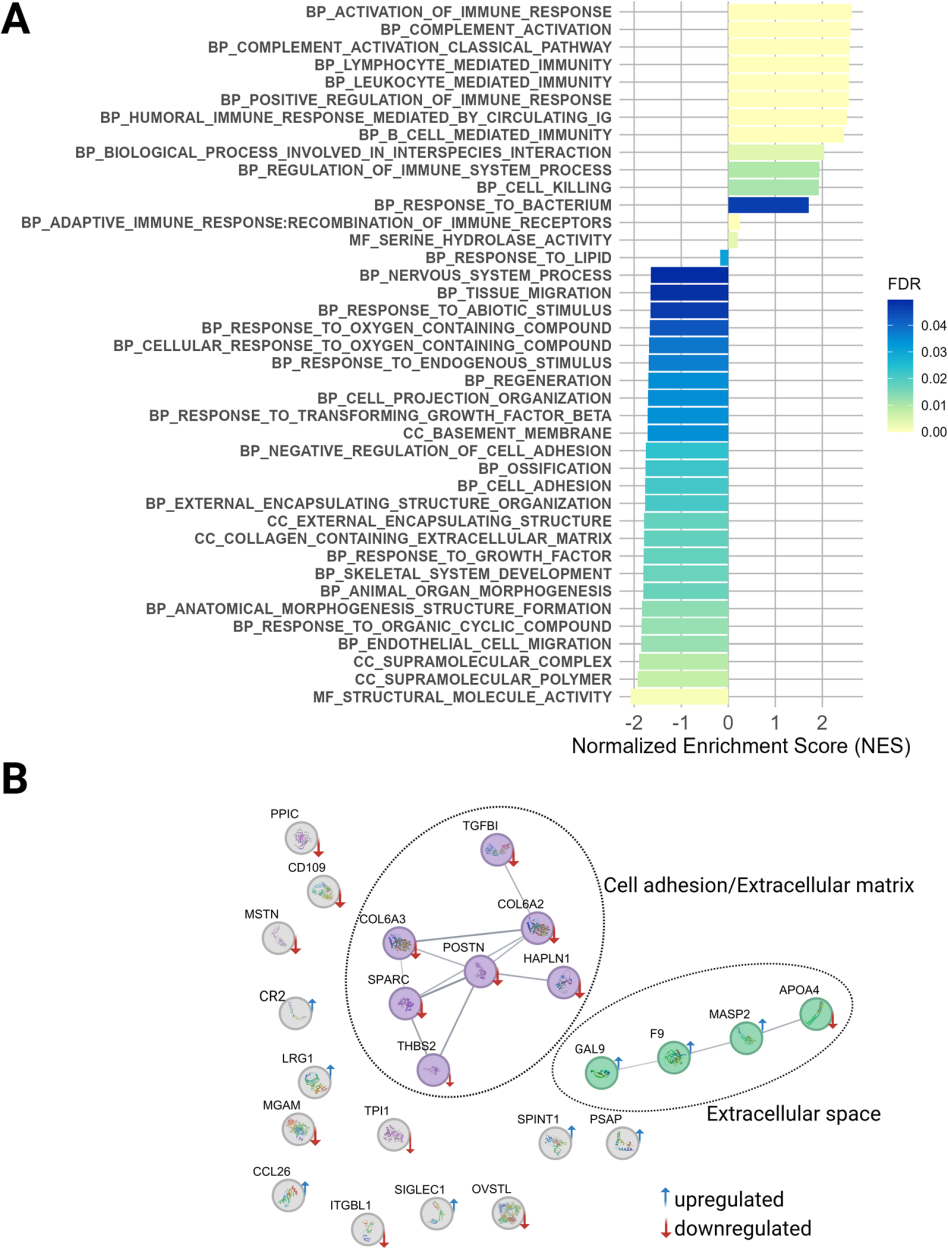
**Functional annotation of differentially expressed proteins. A** Barplot of GSEA-based gene ontology (GO) enrichment analysis (FDR q-value cutoff of 0.05), detailing all terms associated with changes induced by dysbiosis challenge in chicken plasma proteome. Normalized enrichment score (NES) values are mentioned on x-axis, identified categories are mentioned on y-axis. The colors of the bars correspond to the—FDR q-value. Classes of GO terms corresponding to BP: biological process, CC: cellular component, MF: molecular function are presented.** B** Network analysis (PPI) of the differentially expressed proteins**.** Two distinct functional clusters were detected in plasma. Clustering within analyzed protein-coding gene set was based on “evidence” mode at medium confidence (> 0.7), using MCL clustering at 1.5 inflation. The blue and red arrows symbols represent up- and down-regulation, respectively; clusters are marked by dashed lines.

Gene Set Enrichment Analysis (GSEA) [25] was employed to explore the biological functions of proteins affected by dysbiosis. The statistical data from differential abundance testing were used as metrics. The GSEA identified 40 gene ontology (GO) terms that were significantly enriched, reflecting both positive and negative normalized enrichment scores (NES) and highlighting their significant impact on the disease (Figure 5A).

Most of the biological processes enriched in the challenge group were related to activation and regulation of immune responses, including B-cell and leukocyte-mediated immunity, complement activation, as well as response to bacteria and interspecies interactions with other organisms.

In contrast, bioprocesses related to anatomical morphogenesis, cellular organization, adhesion, and response were found among the negatively enriched gene sets.

Additionally, considerable changes were detected in the structures of cellular supramolecular complexes, extracellular matrix (ECM) and basement membrane. Key molecular functions such as serine hydrolase activity and molecular structural activity were also significantly up- and down-regulated, respectively, highlighting alteration in interactions between cells and cellular components.

Other notable bioprocesses affected by the challenge included response to lipids, nervous system processes, and ossification. Detailed information regarding significantly enriched entries can be found in Additional file 2.

To gain a deeper understanding of the physical and functional interactions and regulatory mechanisms of the differentially regulated proteins upon dysbiosis, the STRING database was consulted. In line with the GO analysis, the protein–protein interaction (PPI) analysis revealed that the regulated proteins clustered into functional sub-networks, which are components of the cell adhesion (TGF-β, COL6A2, COL6A3, POSTN, HAPLN1, SPARC, THBS2) and extracellular matrix (ECM) organization (APOA4, MASP2, F9, GAL9) complexes (Figure 5B).

## Discussion

Dysbiosis poses a significant challenge to poultry production systems worldwide, disrupting gut health, compromising growth performance, and impairing nutrient utilization, which underscores the critical need to understand the molecular mechanisms underlying this condition.

The well-established model of dysbiosis employed in this study was designed to mimic subclinical intestinal inflammation and microbial shifts commonly observed in commercial broiler production systems rather than relying on artificial chemical triggers such as dextran sulphate sodium (DSS), lipopolysaccharide (LPS) or feed restriction models that have been used by others [5, 27, 28]. Additionally, the combination of dietary changes (increased NSPs), antibiotic treatment, and oral challenge with opportunistic pathogens is characteristic for cases of human irritable bowel disease (IBD) [9, 11, 29]. Although similar multifactorial models and exact dosing protocols of valid biological relevance have been reported [27, 28, 30, 31], and demonstrated that changes in plasma protein profile in challenged broilers are driven by intensified physiological efforts to control intestinal damage. Furthermore, significant morphologic changes in the intestine align with the findings reported in the literature [5, 32], and were accompanied by increased intestinal permeability. Elevated levels of the gut damage marker ovotransferrin (OVT)—an acute phase protein (APP)—and the higher serum concentrations of iohexol further confirmed the increase in intestinal permeability in challenged birds compared to control group [3, 33, 34].

Proteomics, chiefly, enables sensitive and quantitative analysis of differentially expressed proteins, making it a powerful tool for identifying disease biomarkers [1, 35–37]. In poultry biomarker omics-based research, the use of high-throughput omics methods, such as proteomics, is already generating promising results, such as host proteins detectable in feces [3, 5] or blood [38, 39].

The results of an untargeted MS-based proteomics screen on plasma from broilers upon dysbiosis challenge showed that compared to control animals, dysbiosis significantly changed the abundance of 25 plasma proteins. Enhancement of both innate and adaptive immune responses, including B-cell and leukocyte-mediated immunity, as well as complement activation, aligns with previous findings that gut microbiota imbalances can trigger the immune response [40]. Moreover, findings on compromised fundamental biological functions related to anatomical morphogenesis, cellular organization, adhesion, and response are consistent with the known impact of gut microbiota on cellular integrity and development [41]. Disruptions in cell–cell and cell–matrix interactions, crucial for maintaining gut barrier function and structural stability [42] were affected by the disease given the observed significant alterations in cellular supramolecular complexes, ECM, and basement membrane structures. Additionally, functional enrichment analyses using GSEA and STRING revealed significantly deregulated pathways such as serine hydrolase activity, molecular structural activity, lipid metabolism, nervous system processes, and ossification. Most of these findings may plausibly reflect systemic responses to inflammation or altered nutrient absorption, indicating a systemic impact of dysbiosis on various physiological systems beyond the gastro-intestinal (GI) tract. Nervous system processes are more difficult to directly link to gut dysbiosis, although initially unexpected, may reflect systemic communication via the gut-brain axis in poultry [43]. As reviewed by others, the gut microbiota, microbial metabolites (e.g., SCFAs and indole derivatives), and brain have bidirectional connections, forming an integrated network between the autonomic and enteric nervous systems [35, 43, 44]. While speculative, these findings support a hypothesis of microbiota-driven signaling to the nervous system indicating broader physiological stress or secondary signaling effects requiring further investigation.

An imbalance in the gut microbiome allows certain bacteria to utilize host-derived carbohydrates like sialic acids—integral components of the gut mucus layers—which they incorporate into their surface structures, such as capsules and lipooligosaccharides [45, 46]. This modification helps pathogens evade immune detection by mimicking host cell surfaces and interacting with sialic acid-binding lectins. Therefore, the observed upregulation of sialic acid-binding immunoglobulin-like lectin 1 (SIGLEC1) in chickens affected by dysbiosis is likely to be attributed to complex interactions between host and microbial components. The effect of removal of sialic acids by sialidases is known to contribute to the pathogenesis of necrotic enteritis in broilers [47]. Loss of serine protease inhibitor (SPINT1) function increases intestinal permeability, referred to as “leaky gut” [48], while SPINT-modulated regulation is crucial for preventing conditions such as IBD [49].

Furthermore, challenged birds showed reduced plasma levels of ECM-related plasma peptides, particularly collagen (COL)-associated proteins such as COL6A2 and COL6A3. This likely reflects dysbiosis-induced tissue damage requiring ECM remodeling. The degradation of interstitial collagens is primarily mediated by interstitial collagenases and gelatinases [50], as well as can be modified by exogenous enzymes from gut bacteria. Collagenases produced by *Enterococcus* (*E.*) *faecalis* have been shown to break down collagen in the intestinal wall, contributing to anastomotic leakage [51]. As discovered in animal models, *E. faecalis* activates two pathways for collagen degradation: one via activation of metalloproteinase 9, and the other by converting tissue plasminogen into plasmin, which cleaves collagen and activates matrix metalloproteinases (MMPs). Bacterial enteropathogens employ strategies such as secretion of exotoxins [52], or direct adhesion to the host intestinal epithelium and ECM molecules leading to their exposure into the gut lumen [32, 53]. Adherence to ECM components or intestinal epithelium facilitates passage of enterotoxins to deeper tissue layers [53, 54]. Pathogenic bacteria like *E. coli* [55], *Mycobacterium* spp. [56], and *Enterococcus* spp. [57] express surface proteins that bind to ECM components such as collagen, fibronectin, and laminin, facilitating host colonization.

Invasion of enterocytes by *Eimeria* spp. cause destruction of host mucosal cells, resulting in increased permeability, plasma protein leakage, impaired digestion, and reduced absorptive surface area [53, 54]. Transcriptome analysis of chicken cecal epithelia during coccidiosis has shown up-regulation of MMP and down-regulation of genes encoding metabolic enzymes, membrane components, and some transporters [58]. Damage to epithelial cells by coccidia invasion may allow exposure of ECM proteins, making them accessible to pathogenic microorganisms [53, 59].

Under the condition of compromised integrity of the epithelial monolayer, rapid and efficient restoration of the epithelial barrier is crucial to maintaining intestinal homeostasis and preventing uncontrolled inflammatory responses [60]. Derkacz et al. highlighted that sustained and excessive inflammatory responses in the intestinal tissue lead to progressive alterations in the structure and function of components such as the ECM exacerbating inflammatory responses [42]. This is evidenced by the upregulation of complement pathway components, chemokine, and an antimicrobial peptide.

Upregulation of gallinacins (GALs) in response to challenge in broilers additionally highlights a critical role in immune defense in the context of intestinal inflammation (Figure 5B). GAL-9, a beta-defensin antimicrobial peptide, plays a crucial role in the innate immune system of chickens, exhibiting significant antimicrobial activity against pathogens such as *E. coli* and Salmonella serovars, as confirmed by studies in silico [61, 62]. The elevated levels of GAL-9 detected in the present study confirm its defensive function against bacterial infections.

In human and murine models, leucine-rich alpha-2 glycoprotein 1 (LRG-1) has recently emerged as a useful biomarker of chronic inflammatory bowel disease, reflecting the disease activity [63, 64]. LRG-1 has been shown to participate in cell adhesion, migration, and survival [65]; its expression was detected i.a. in intestinal epithelial cells. We found increased LRG-1 levels in chicken blood plasma. This aligns with the results of our previous study on the proteome response to necrotic enteritis in broilers [66].

Increased plasma levels of prosaposin (PSAP), a precursor of saposins-lysosomal proteins that are essential for the activation of hydrolases involved in sphingolipid metabolism, were discovered [67]. In the context of gut inflammation, PSAP exhibits potent antimicrobial properties and modulates lysosomal functions, activating macrophages, and contributing to the innate immune response. Moreover, an in vitro study has shown a contribution of PSAP in maintaining cellular CoQ10 levels and forming tight junctions in the gastrointestinal tract [68].

The current findings align with previous studies on necrotic enteritis in broilers, where immune-related proteins in the blood were also upregulated, reflecting an activated immune response [69]. While lesion scoring estimates *C. perfringens*-induced damage, we assessed dysbiosis through intestinal permeability (serum iohexol quantification) and fecal OVT levels. These results highlight how gut health disruptions, regardless of cause, impact plasma protein profiles, suggesting compromised tissue integrity and remodeling. Intestinal permeability is tightly regulated by crypt-associated pathways monitored by mesenchymal cells, which interact with the ECM and respond to inflammatory and microbial cues [70]. Given the continuous communication with the intestinal epithelium and interaction with immune cells within the lamina propria, mesenchymal cells are able to detect changes in their microenvironment, recognize microbial signals, and thus get activated by the inflammatory conditions drawn by innate immune cells [60].

Adding to this, dynamic ECM protein networks, present in all tissues, are synthesized by fibroblasts [42, 60, 71]. Therefore, previous and present proteomics results allow hypothesizing that significant changes in mesenchymal cell physiology induced by intestinal inflammation or pathogen intervention could lead to the downregulation of ECM components.

While this study demonstrates the utility of plasma proteomics for identifying candidate blood-based biomarkers of intestinal dysbiosis in broilers, several limitations must be acknowledged. Firstly, the absence of concurrent microbiome profiling limits direct correlation between microbial shifts and proteomic changes. Secondly, the use of antibiotics to induce dysbiosis introduces potential confounding effect, as antibiotics may directly affect host metabolism, immune signaling, or mitochondrial function, independently of microbial imbalance per se. Lastly, regardless of origin, systemic inflammation can alter plasma protein profiles, modifying normal physiological functions of the proteome [72, 73], thus some proteomic changes may reflect nonspecific inflammation rather than gut-specific responses.

Importantly, translating MS-based proteomic findings into field diagnostics might pose a notable challenge due to technical complexity and resource demands, limiting routine use in large-scale poultry settings. Targeted approaches, such as ELISA or selected reaction monitoring (SRM), will be essential for validating and applying candidate biomarkers in practice. Undoubtedly, further validation across different enteric diseases, field cases, large-scale trials, or in vitro assays is needed to confirm their reliability as general gut health biomarkers in poultry.

In conclusion, the present findings suggest that the observed plasma proteomic alterations in the dysbiosis model reflect the host's systemic response to restore gut homeostasis, involving immune activation and structural tissue remodeling. While direct evidence on ECM reduction in poultry dysbiosis is limited, further investigation into the role of ECM in intestinal integrity and protein metabolism could enhance understanding of disease mechanisms. Candidate biomarkers such as GAL-9, LRG-1, and PSAP show promise of impaired gut health but require further validation to assess their specificity, sensitivity, and early predictive value. Noteworthy, reported findings are preliminary and should be considered hypothesis-generating.

## Supplementary Information

**Additional file 1. List of proteins (*n* = 388) identified and quantified with DIA, with corresponding differential abundance analysis in Challenged vs Ctrl group.** Columns from left to right contain protein and gene IDs,protein name, *p*-value, adjusted (adj.) *p*-values, log(FC), results of t-statistic, significance label, log_2_ LFQ expression values.

**Additional file 2. Gene Set Enrichment Analysis (GSEA) for quantified protein-coding genes**. Columns from left to right represent classes of gene ontology (GO) terms (CC: cellular component, BP: biological process, MF: molecular function), number of genes in the gene set after filtering (SIZE), Enrichment Score (ES), normalized enrichment score (NES), nominal *p*-value (NOM *p-*val), and false discovery rate (FDR q-val).

## Data Availability

The mass spectrometry proteomics data have been deposited to the ProteomeXchange Consortium via the PRIDE [74] partner repository with the dataset identifier PXD056546 (Username: reviewer_pxd056546@ebi.ac.uk and Password: DV2LINNIyVsS).

## Abbreviations

AGP: antimicrobial growth promoter
ECM: extracellular matrix
BW: body weight
CFU: colony forming unit
OVT: ovotransferrin
DIA: Data Independent Acquisition
DTT: dithiothreitol
FA: formic acid
ACN: acetonitrile
PPI: protein–protein interaction
HPLC–MS: high performance liquid chromatography–mass spectrometry
PCA: principal component analysis
RT: room temperature
TEAB: triethylammonium bicarbonate
SDS: sodium dodecyl sulphate
iRT: Indexed Retention Time peptides
MBR: match between runs
FC: fold change
GSEA: Gene Set Enrichment Analysis
GO: gene ontology
BP: biological process
CC: cellular components
MF: molecular function
MCL: Markov Cluster Algorithm
APP: acute phase protein
CCL26: chemokine 26
CR2: complement receptor 2
CKM: creatine kinase M-type
GAL9: gallinacin 9
APOA4: apolipoprotein 4
NES: normalized enrichment score
ITGBL1: integrin subunit beta 1
THBS2: thrombospondin 2
HAPLN1: hyaluronan and proteoglycan link protein 1
SPARC: secreted protein acidic and cysteine rich
COL6A2: collagen α-2(VI) chain
COL6A3: collagen α-3(VI) chain
TGFBI: transforming growth factor-β-induced protein Ig-h3
MASP2: mannan binding lectin serine peptidase 2
F9: coagulation factor IX
SIGLEC1: sialic-acid-binding immunoglobulin-like lectin 1
SPINT1: serine peptidase inhibitor 1
MMP: matrix metalloproteinases
DSS: dextran sulphate sodium
LPS: lipopolysaccharide
IBD: inflammatory bowel disease
PSAP: prosaposin
LRG1: leucine-rich alpha-2 glycoprotein
ELISA: enzyme-linked immunosorbent assay
SRM: selected reaction monitoring
